# FLYCOP: metabolic modeling-based analysis and engineering microbial communities

**DOI:** 10.1093/bioinformatics/bty561

**Published:** 2018-09-08

**Authors:** Beatriz García-Jiménez, José Luis García, Juan Nogales

**Affiliations:** 1 Department of Systems Biology, Centro Nacional de Biotecnología, Consejo Superior de Investigaciones Científicas (CNB-CSIC), 28049 Madrid, Spain; 2 Microbial and Plant Biotechnology Department, Centro de Investigaciones Biológicas (CIB-CSIC), 28040 Madrid, Spain; 3 Applied System Biology and Synthetic Biology Department, Institute for Integrative Systems Biology (I2Sysbio-CSIC-UV), 46980 Paterna, Spain

## Abstract

**Motivation:**

Synthetic microbial communities begin to be considered as promising multicellular biocatalysts having a large potential to replace engineered single strains in biotechnology applications, in pharmaceutical, chemical and living architecture sectors. In contrast to single strain engineering, the effective and high-throughput analysis and engineering of microbial consortia face the lack of knowledge, tools and well-defined workflows. This manuscript contributes to fill this important gap with a framework, called FLYCOP (FLexible sYnthetic Consortium OPtimization), which contributes to microbial consortia modeling and engineering, while improving the knowledge about how these communities work. FLYCOP selects the best consortium configuration to optimize a given goal, among multiple and diverse configurations, in a flexible way, taking temporal changes in metabolite concentrations into account.

**Results:**

In contrast to previous systems optimizing microbial consortia, FLYCOP has novel characteristics to face up to new problems, to represent additional features and to analyze events influencing the consortia behavior. In this manuscript, FLYCOP optimizes a *Synechococcus elongatus-Pseudomonas putida* consortium to produce the maximum amount of bio-plastic (PHA, polyhydroxyalkanoate), and highlights the influence of metabolites exchange dynamics in a four auxotrophic *Escherichia coli* consortium with parallel growth. FLYCOP can also provide an explanation about biological evolution driving evolutionary engineering endeavors by describing why and how heterogeneous populations emerge from monoclonal ones.

**Availability and implementation:**

Code reproducing the study cases described in this manuscript are available on-line: https://github.com/beatrizgj/FLYCOP

**Supplementary information:**

Supplementary data are available at *Bioinformatics* online.

## 1 Introduction

### 1.1 Consortia versus single strain design

The design and engineering of microbial species has been successfully applied during the last decades to produce compounds of environmental, industrial and health interest; increasing the efficiency of natural procedures, reducing cost and making possible even new transformations (Dvořák *et al.*, 2017; Khalil and Collins, 2010; Kumar *et al.*, 2016*;*Revuelta *et al.*, 2016). Approaching biotechnology transformations in terms of substrates and products complexity, biotechnology procedures have gone from single transformations until complex ones, going from low complexity substrates and products (i.e. from glucose to ethanol), through medium complexity transformations (i.e. from cellulose to antibiotics), until the current moment, where high complex molecules including waste or pollutant compounds (such as plastics or lignin) are expected to be degraded/transformed in assorted high-value products, such as flavonoids, vitamins, isoprenoids or steroids (Hansen *et al.*, 2017*;*Vitorino and Bessa, 2017). All the previous stages of the biotechnological transformations have been solved engineering single strains (Chae *et al.*, 2017; Gustavsson and Lee, 2016).

Aimed by the multiple advantages provided by microbial communities, nowadays cutting-edge biotechnological approaches begin to propose to microbial consortia as more effective strategy in order to solve these challenging high complex biotransformations (Brenner *et al.*, 2008; Cavaliere *et al.*, 2017; Foo *et al.*, 2017; Zhang and Wang, 2016).

In Nature, free microbes live in communities, establishing complex relationships with other species, and rarely isolated as most of engineered microbes do; therefore, engineering microbial consortia will be closer to natural and physiological behavior than isolated strains. Other advantage of microbial consortia is they can carry out additional or new functions, such as synthesizing more complex molecules (e.g. a costly extracellular enzyme). This complex compound would be the product of a cooperative effort of several strains, in contrast to a single strain producing a metabolite associated to growth.

Pathway modularization is also a great advantage of communities (Eng and Borenstein, 2016; Julien-Laferrière *et al.*, 2016). Diverse metabolic functions could be split and distributed among different strains in the community. Thus, a set of highly adapted and specialized strains could carry out a particular metabolic function each within the whole pathway designed to solve the biotechnological task. It implies less genetic transformation per strain, with lower technical complexity, increasing the success likelihood. Moreover, a microbial community could increase the efficiency and bioproduction performance in the biotechnological transformation of the input compound. It could be achieved by a suitable utilization of different substrates, or allowing the synthesis of several products by different strains, or avoiding the accumulation of intermediate metabolites by adjusting the relative subpopulation sizes in the community. Finally, microbial consortia provide robustness against internal metabolic and environmental stresses, which are decreased by spatial segregation that avoids undesired interferences of toxic intermediate products.

Despite all these advantages of applying synthetic microbial communities for biotechnological transformations, there are several unresolved challenges (Zhang and Wang, 2016) to take into account to design a synthetic consortium. The most important one is to determine the conditions that allow co-culture and growth compatibility of different strains, given that the engineered strains usually are optimized for metabolite production rather than cooperative growing as in natural communities. Another challenge is to select the appropriate intermediate metabolites which could be transported through membranes between different strains in the consortia.

To design those microbial consortia for biotechnological applications, we need to increase the knowledge about how a community works at systems level, and also to increase the available tools to design and construct microbial consortia, both at computational and experimental levels. Thus, this work contributes with a computational framework, called FLYCOP (FLexible sYnthetic Consortium Optimization), to improve the understanding of the metabolic behavior of microbial consortia and to automatize the modeling and computational design of those communities.

### 1.2 Metabolic modeling approaches

At single strain level, genome-scale metabolic reconstructions are organism specific knowledge bases. Such reconstructions are developed systematically through the integration of genome annotation, omic dataset and biological knowledge available for the target species at the time of reconstruction. They can be further transformed into computational models enabling the quantitative prediction of phenotypic states in terms of fluxes through individual reactions (Bordbar *et al.*, 2014). Constraints Based Reconstruction and Analysis (COBRA) (Ebrahim *et al.*, 2013; Schellenberger *et al.*, 2011) methods have become popular for analyzing metabolic models. COBRA methods and the large array of strain-design algorithms available are commonly apply for single strain *in silico* design, systems metabolic engineering and optimization.

At community level, there are several tools from a descriptive point of view that check the behavior of a particular microbial configuration. However, current modeling approaches at community level neither allow optimization nor design. Perez-Garcia *et al.* (2016) categorized microbial consortia modeling descriptive approaches within the context of stoichiometric metabolic models. From simpler to more complex, the groups are: a) lumped network, b) compartment per guild (multi-compartment), c) bi-level optimization and d) hybrid (or Dynamic-Stoichiometric Metabolic Network). Although each approach is recommended for modeling different kind of interactions and scenarios, the last one provides with the highest capabilities for designing complex conditions in microbial communities and with the flexibility for modeling a wide range of microbial consortia. Based on Perez-Garcia *et al.* (2016) criteria, that hybrid approach is the most suitable for engineering microbial consortia, because it is the optimal approach to quantify: 1) inter-species interactions, 2) temporal changes in metabolites concentration and 3) physiology at community level, which are properties required for consortia design and optimization. Additionally, this hybrid approach is suggested as the more promising for communities with low species richness, a similar scenario to that expected in synthetic microbial consortia designs for biotechnological transformations. So it is the best descriptive approach, where multiple scenarios could be modeled and multiple configurations could be represented and optimized using our system.

There are two main available tools classified as hybrid approaches: Microbial Community Modeller (MCM) (Louca and Doebeli, 2015) which only has been tested in an *E.coli* community, without combining different species as a consortium usually includes; and COMETS (Harcombe *et al.*, 2014) which has been successfully applied in the modeling of multiple species communities, and even engineered, microbial communities. BacArena (Bauer *et al.*, 2017) is an alternative recent method for describing microbial consortia, with an individual agent-based approach versus population modeling as COMETS considers. Although it could be an alternative to COMETS, it was not explicitly classified as a dynamic approach, and it does not allow all strains to grow in the same cell, limiting its application to simplified scenarios with unknown experimental diffusion parameters. Therefore, COMETS was selected as our computational tool for describing consortia behavior.

Further than a descriptive view of microbial consortia, methods which design and optimize them are scarce and mainly ad-hoc for particular applications. There are tools for designing and optimizing single strains for metabolic engineering, such as CAMEO (Cardoso *et al.*, 2018). However, their corresponding design tools at consortia level represent a current challenge in microbial communities, where our new system will contribute.

Given the current challenges in microbial consortia design, the objective of this manuscript is to provide a computational framework, called FLYCOP, to automatically design and optimize microbial consortia given a personalized goal.

FLYCOP contributions are multiple and assorted. Firstly, its wide flexibility in several aspects which allow its use under very diverse microbial scenarios with distinct goals. Second, FLYCOP helps to understand how microbial communities work at systems level. Besides, it could save resources and time, avoiding or reducing chemical optimization or trial-and-error attempts, by the automatization of manual checking of different consortia configuration. FLYCOP also advances in ‘predicting the composition of a microbial community in a given environment’, recently defined as a cross-cutting task (Chan *et al.*, 2017). FLYCOP finally contributes in helping to define the unknown objective function of evolving communities, which has been proposed as a challenge (Gottstein *et al.*, 2016).

Next sections describe FLYCOP in depth, compare it with previous related systems and illustrate FLYCOP’s broad applicability by addressing several cases of study of automatic designs of microbial communities: a *de-novo* synthetic consortium, a multiple cross-feeding preserving stability case and engineering evolution of monoclonal communities.

## 2 Material and methods

### 2.1 FLYCOP

FLYCOP (FLexible sYnthetic Consortium OPtimization) is a framework for the *in silico* modeling of microbial communities, exploring multiple consortium configurations in an automatic and guided way, optimizing a customized consortium goal. FLYCOP allows the integration, analysis and optimization of specific genome-scale metabolic models (GEMs) describing partners in the community.

The design of a microbial consortium addressing a particular biotechnological task requires of thousands of possible consortium configurations and multiple criteria to evaluate, making unfeasible to carry out an exhaustive exploration and manual evaluation of the different configurations. Thus, FLYCOP was designed to avoid the trial-and-error of multiple random consortium configurations.

Therefore, rather than tuning each control point one by one, FLYCOP tackles this multiple objective problem with an optimization approach, through a process called stochastic local search (Hoos and Stützle, 2004). Thus, this kind of search procedure guides the exploration through the optimal microbial consortium or near solutions.

**Fig. 1.**
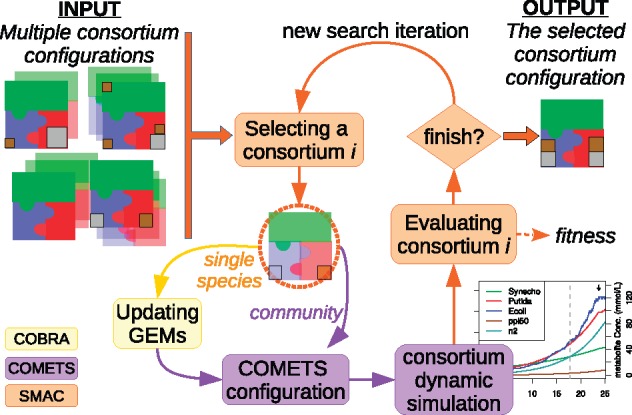
FLYCOP diagram. Follow the arrows from input (top left corner) to output (top right corner) in a counter-clockwise direction. In this exemplifying consortium optimization by FLYCOP, in each puzzle, the three colored big pieces (green, blue and red) represents three different microbial strains in the consortium. The different brown and gray little squares represent two distinct metabolites whose secretion rate should be optimized and distributed among the consortium members. Thus, in the putative input configurations there are different metabolite secretion rates and distributions, and the output puzzle represents the best consortium configuration found by FLYCOP, maximizing the secretion

In a simplified view, FLYCOP takes many consortium configurations as input and returns just one configuration as output, i.e. the best found. Figure 1 outlines our FLYCOP algorithm, including the following steps: 1) Updating metabolic models in COBRA, computing and/or changing bounds in the particular secretion reactions involved in tuning the metabolites exchange (cross-feeding, removing or overproducing metabolic compounds); 2) Establishing dynamic and community COMETS parameters (for example, initial biomass of each strain) in its layout configuration file; 3) Simulating a consortium dynamic evolution of different strains; 4) Computing fitness (i.e. quality measure); and 5) Checking and updating for new search iteration (going back to step 1) by SMAC (Sequential Model-based Algorithm Configuration) (Hutter *et al.*, 2011) or to finishing the optimization process when the maximum number of cycles is reached.

The input to FLYCOP are: GEMs of the microbial strains in the community, parameters describing the consortium configuration and their range of suitable values, and a fitness function. The consortium configuration includes several parameters with multiple values to check for each one, while the assessment score will determine the quality of each consortium over an iterative procedure, evaluating how a specific combination of values affect the consortium behavior. A fitness or evaluation function is required to compare the different solutions and to describe the optimization objective—this is a key point for the FLYCOP optimization. The fitness function must be meticulously designed according to the consortium optimization goal. In a multi-objective case, a weighted sum of individual objectives should be defined.

FLYCOP output is a customized consortium configuration optimizing a given particular objective defined in a fitness function. Each consortium configuration is described by a set of parameter values. Those values are optimized with stochastic local search, according to the fitness function. The fitness value of each configuration is computed as the mean of several COMETS runs with the same configuration, due to the random nature of COMETS (varying the order of execution of the different models growing in the same cell space, mimicking a real scenario). In FLYCOP, all consortium simulations are carried out in a single spatial point (i.e. a 1 by 1 grid). Besides, FLYCOP allows to include physiological constraints that reduce the computable space, i.e. consortium configurations to be rated by FLYCOP.

FLYCOP was designed as a dynamic integration of different technologies. Thus, in every iteration, FLYCOP a) updates single metabolic models with COBRA (Schellenberger *et al.*, 2011), b) performs the consortium simulation with COMETS (Harcombe *et al.*, 2014) and c) selects and evaluates candidate consortium configuration with SMAC (Hutter *et al.*, 2011). Supplementary Material includes a brief description of each technology.

In FLYCOP, we have chosen the SMAC framework, an iterated local search algorithm applied to a parameter configuration space, to make possible the selection of the most suitable combination of values of a given list of parameters from a set of given values. The output configuration provided by SMAC is typically applied to problems such as Boolean satisfiability problems (SAT), probabilistic reasoning, automatic planning, or even protein folding. Because of its success in solving problems in assorted domains, it was considered a suitable approach to optimize microbial consortia.

Although FLYCOP returns a unique solution from the local search optimization, there is a number of alternative solutions with high fitness values (closer to the highest one) that could be of interest. Therefore, a data mining analysis is automatically applied to report a summary of the FLYCOP evaluated configurations, and the description of the best ranked ones.

### 2.2 Flexibility: multiple applications categories

As a general overview, FLYCOP is applied to design microbial consortia, optimizing a given goal, through defining their configuration. It could be instantiated in multiple and assorted specific FLYCOP applications, such as simulating different scenarios before *in vivo* experiments; defining medium composition, detecting limiting nutrients; discovering the biological metric optimized in an evolutionary process; optimizing cross-feeding relationships; optimizing strain ratios in the consortium; optimizing pathway fragmentation among several strains (first, grouping genes or reactions and second, distributing these groups among strains in the consortium); etc. From other point of view, FLYCOP could increase the knowledge about microbial communities solving some questions such as how to preserve stability through the configuration of cross-feeding rates and strain ratios; how to maximize pollutant cleaning through assigning cleaning tasks to different strains in the consortium; or to describe why or how heterogeneous populations emerge from monoclonal ones.

FLYCOP proposes a very generic and flexible approach, applicable to many designs of microbial consortia characterized by different combinations of strain compositions, goals and parameters to configure, that are categorized and summarized in Table 1. It is remarkable that new categories of optimization goal and configurable parameters could be defined and several ones combined. The ‘configurable consortium parameter’ category represents the prediction output. A priori whatever mixture of categories is feasible for FLYCOP, although we recommend taking physiological conditions into account to define meaningful applications. The first three categories (strain ratios, cross-feeding rates and co-metabolism) represent the most common parameters required to configure when a synthetic consortium is designed. The ATP maintenance coefficient category has been included because it has been reported as a high influencer over the community composition, especially for small GR (Koch *et al.*, 2016).

**Table 1.** Categorization of possible FLYCOP applications, according to different sub-types of strain composition, optimization goal and configurable consortium parameters

**Strain composition**
2 homogeneous/monoclonal strains (*phenotypic heterogeneity)*
2 heterogeneous strains *(genotypic and phenotypic heterogeneity)*
> 2 strains *(homo- or hetero-)*
**Optimization goal**
Maximize Growth Rate (GR) *(not limited carbon source)*
Maximize yield *(biomass per carbon unit, limited carbon source)*
Maximize production of metabolite of interest
Minimize degradation time of contaminant metabolite
Minimize time to reach stationary phase
Minimize time to exhaust resources
Maximize parallel growth/stability
**Configurable consortium parameters**
Strains ratio
Cross-feeding rates
Co-metabolism *(carbon sources ratio, when >1 carbon sources)*
Medium composition
Initial carbon source concentration
Pathway fragmentation and consortia partner selection
Aerobic-anaerobic switching time
ATP maintenance coefficient

## 3 Results

The first subsection explains a detailed comparison of FLYCOP with other consortium optimization methods. The last three subsections describe three distinct FLYCOP applications, where specific and different cases show the flexibility of our framework and also validate our computational optimization with already published *in vivo* studies.

### 3.1 Comparison consortia optimization methods

Table 2 collects a descriptive comparison of FLYCOP with other methods optimizing microbial communities. The main common differences between FLYCOP and the other consortia engineering and optimization methods are: a) FLYCOP designs optimized consortia automatically by evaluating different configurations and then selecting the best one, in a reasonable time; b) the active design versus just a descriptive approach, which often requires many experimental values, contrasting with FLYCOP, which could suggest some of those experimental parameters; c) it is based on the hybrid approach, being the most complex one, of high interest and recommended for synthetic engineered species, allowing FLYCOP to describe changes in metabolite concentration in the medium, which is a not an available capability in bi-level or lumped approaches.

**Table 2.** Summary comparison engineering and optimization consortium methods, classified according to optimization goal

Optimization goal	**Flexible** (FLYCOP)	**Community parameter** (d-OptCom) (Zomorrodi *et al.*, 2014)	**Stability** (SteadyCom) (Chan *et al.*, 2017)	**Pathway distribution** (MultiPlus) (Julien-Laferrière *et al.*, 2016)
**Descriptive metabolic modeling**	Hybrid	Bi-level	Bi-level	Lumped
**Stoichiometric knowledge**	Yes	Yes	Yes	No
**Optimization approach**	Local search (SMAC)	Global search (BARON)	Iterative LP	Dynamic programming
**FBA solver complexity**	Linear	Bilinear	Linear	–
Multi objective (*1)	Yes	Limited	No	Limited
**Flexible objective**	Yes	Yes	No	No
**No. strains**	Small	Small	High	Small
**Kinetics parameter**	No (optional)	Yes	Yes (optional)	No
**New (LP) constraints**	No	Yes	Yes	No
**Data Analysis support**	Yes	No	No	No
**Software availability**	Yes	No	Yes	Yes

*Notes*: *1: FLYCOP allows several objectives at community level; d-OptCom, 1 single strain level + 1 community level objectives; MultiPlus, 2 fix objectives: minimizing reactions and minimizing exchanged metabolites.

Summarizing Table 2, the most relevant FLYCOP contributions are: 1) Dynamic (hybrid) versus static (bi-level/lumped) as descriptive metabolic modeling, which means to design and configure the community versus just to describe it; 2) Flexible optimization objective (different ones), in contrast to a fixed objective in each method; 3) Multiple and assorted uses cases; 4) Multi-objective, with FLYCOP combining several objectives in a unique fitness function; 5) Not requiring to define new equations to constrain at low level (i.e. linear programming); and 6) Integrated data analysis. A detailed comparison is available in Supplementary Material.

In addition, FLYCOP can maximize one metric and minimize another one at once, defining the desired fitness function, such as maximize biomass and minimize time. FLYCOP model mutations are not limited to rates in individual models, but they could also be population properties, as ratio of strains, or carbon sources ratios.

### 3.2 Case study 1: Step-by-step synthetic consortium design with FLYCOP: *S.elongatus-P.putida*

**Fig. 2.**
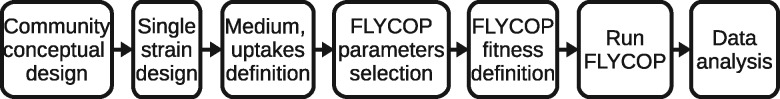
Step-by-step workflow for designing and optimizing a microbial consortium with FLYCOP

Following the steps in Figure 2, we describe how to apply FLYCOP to design and optimize a new synthetic microbial consortium.

**1. Community conceptual design**. First, the conceptual design of the consortium must be defined, selecting the strains within it and the suitable cross-feeding relationships that make the desired biotechnological task feasible. In this case study, as a FLYCOP proof of concept, we design and optimize a *S.elongatus-P.putida* consortium where the former feeds with sucrose to the latter, which produces bio-plastic (polyhydroxyalkanoate, PHA) under ammonium limiting conditions. FLYCOP determines the optimal values of sucrose to be secreted by the cyanobacterium (it means the percentage of fixed carbons that *S.elongatus* dedicates to produce sucrose rather than growing itself), strains ratio (initial biomasses) and NH_4_ concentration in the culture medium. This case study covers several categories of FLYCOP flexibility applications (see Table 1): it is a multi-strain consortium with 2 heterogeneous strains; the optimization goal is to maximize the production of a metabolite of interest (PHA); and it involves several categories of configurable parameters: strain ratio, cross-feeding rate (sucrose) and culture medium composition (NH_4_ concentration). Besides, this is a synthetic consortium susceptible to be validated in a wet lab.

**2. Single strains design**. The base genome-scale models of the selected bacterial species should be modified for co-living in the consortium; adding, removing or modulating the required reactions and their bounds. Table 3 summarizes how those steps are instantiated in the current case study.

**Table 3.** Definition steps 1–6 (identified in Fig. 2) of *S.elongatus-P.putida* synthetic consortium design with FLYCOP

Step	Current case study
1	*S.elongatus (synecho)* --[sucrose]→ *P.putida (KT)*→ PHA
2	*i*JB785 (Broddrick *et al.*, 2016)^a^: co-overexpress *cscB* & *sps*^b^
	*i*JN1411 (Nogales *et al.*, 2017)^c^: +invertase, -NTRARx, NTRIR2x^d^
3	BG11 medium (Rippka and Deruelles, 1979), NH_4_ concentration defined by FLYCOP output
	KT sucrose uptake: 1/2 glucose (3.1 mmol/gDWh^−1^)^e^
	KT PHA^f^ uptake: 1.83 mmol/gDWh^−1^^g^
4	Sucrose secretion rate: from 10 to 80; default: 30%^h^
	Initial biomass synecho: from 0.5 to 2; default: 2 g/L^i^
	Initial biomass KT: from 0.02 to 0.2; default 0.1 g/L^j^
	Concentration of NH_4_: from 0.5 to 15; default 7 mM^k^
5	Fitness=maximizing accumulated PHA in 100 hours
6	sucrPer=40%, synecho=2 g/L, KT=0.2 g/L and NH_4_=0.5 mM; PHA=22.43 mM in 100 hours

a Updated including more detailed sucrose and lipids metabolisms as well as by removing minor bugs such as the requirement for leucine for growth.

b To secrete sucrose under salt stress (Duan *et al.*, 2016).

c The latest and more complete *P.putida* GEM available, which include around 3000 reactions.

d To induce nitrogen limitation conditions only in *P.putida KT2*440 but not in *S.elongatus* growing in BG11 mineral medium which contains nitrate as nitrogen source; nitrate assimilatory system (codified by PP_1703-06 genes) was removed.

e Del Castillo *et al.* (2007).

f Since *i*JN1411 is able to synthesize a large number of PHA monomers (up to 27), we considered the C8 monomer as model PHA.

g The maximum PHA production rate of KT using sucrose as carbon source was computed in COBRA under the above constraints and the PHA production as objective function.

h max. 80% (Ducat *et al.*, 2012).

i It lets a growing margin until the 3.5 g/L when the sucrose secretion is induced with NaCl in (Duan *et al.*, 2016).

j The default value of the feeding strain initial biomass (synecho) is at least one order of magnitude higher than the eating strain biomass (KT). Values definition based on the feasible amount of biomass obtained from a colony after a typical incubation period.

k The maximum value comes from M63 medium, a common minimal medium used for KT growth; while the lower limit is below of the NH_4_ concentration used for PHA production under nitrogen limiting conditions, e.g. 1.5 mM (Prieto *et al.*, 2016).

**3. Culture medium composition and uptake of metabolites definition**. A culture medium composition where the assorted strains within the consortium can live together must be defined. Additionally, a literature search or COBRA model checking is required to define the maximum uptake of carbon source (and maybe other limiting nutrients such as O_2_) and metabolite secreting rates.

**4. Selecting FLYCOP parameters and range of values**. It represents the variables (from categories defined in Table 1) whose values combinations characterize each evaluated consortium. Discrete values are defined instead of continuous ones, to reduce the optimization problem complexity, and to prevent that FLYCOP explores very close configurations.

**5. Defining FLYCOP optimization goal and fitness function**. A function to optimize must be defined, according to the categories described in Table 1. Other important point to define is where to compute the fitness (exponential or stationary phase, in a unique point or an average, etc.), depending on the consortium goal.

**6 and 7. Run and FLYCOP-supported data analysis**. A FLYCOP run must explore enough different configurations (usually around 500) to converge a solution and to get a robust data analysis of the evaluated configurations. FLYCOP provides different resources for robustness, sensitivity and data analysis support: scatterplots showing the distribution of the explored values by each parameter; correlation and ellipse plots; growth curves of all distinct evaluated consortia; tables with all the evaluated configurations, including parameter values, fitness and some other interesting metrics (such as the metabolite concentrations). Those metrics allow further data analysis, such as decision rules identifying the highest X% of best solutions, or the subset of the most influencing parameters in discriminating high from low fitness consortium configurations.

In the current case study, FLYCOP found a robust configuration, producing the highest PHA (∼22 mM) in 100 hours. That configuration shares parameter values with the best 5% of solutions (sucrose percentage = 40%, synecho = 2 g/L (highest value) and increasing NH_4_, from 0.5 to 3.5 mM).

**Fig. 3.**
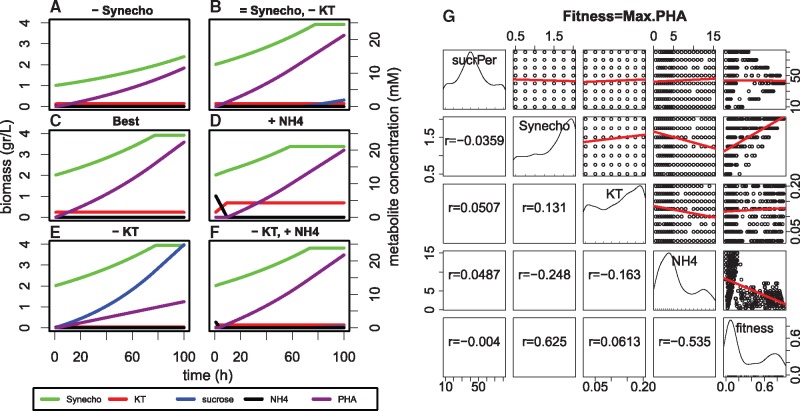
FLYCOP results *S.elongatus-P.putida* consortium producing PHA. (**A–F**) Consortium growth curves with different configurations explored by FLYCOP, corresponding to (% sucrose secretion by Synechococcus, initial biomasses of cyanobacteria and *KT*, NH_4_ initial concentration): A) 40, 1, 0.1, 0.5; B) 40, 2, 0.1, 0.5; C) 40, 2, 0.2, 0.5; D) 40, 2, 0.2, 7; E) 40, 2, 0.02, 0.5; F) 40, 2, 0.02, 2. Each one represents one modification (or two in B and F) versus the best configuration (panel C). **(G)** Sensitivity analysis with scatter-plots (upper triangle) and correlations (lower triangle) between fitness (last column and row) and consortium parameters (remaining columns and rows). In each individual plot, X-axis corresponds to column variable and Y-axis to row variable [for example, the upper right corner plot represents fitness (X) versus %sucrose (Y)]. Linear-regression lines in red. Main diagonal represents histograms of the parameter values

Figure 3G shows the distribution of the different explored configurations (parameter values) and their correlations with the fitness and also between the different pairs of variables. The best fitness correlation (0.62) points out that the higher the initial *S.elongatus* biomass is, the higher the fitness (i.e. PHA production) is. It makes physiological sense, because a lower sucrose-producer biomass means a lower sucrose concentration available to *P.putida* to transform it on less PHA (Fig. 3A). This fact is related to another clear conclusion: the secretion of 40% sucrose by the cyanobacteria, drawn by the arrow shape in the percentage of sucrose versus fitness (upper right corner in Fig. 3G). Moreover, the above-mentioned conclusion is an interesting prediction, because the published secretion rates are around 80% (Ducat *et al.*, 2012), thus FLYCOP suggests that the design of synthetic and stable consortia should be based on sucrose overproducer cyanobacteria secreting less sucrose but over longer time.

The second most relevant fitness correlation is with NH_4_, being inverse in this case (−0.54), indicating that the higher the initial ammonium concentration is, the lower the fitness is. This fact is in agreement with literature, where a low NH_4_ concentration is associated with PHA production (Prieto *et al.*, 2016). Thus, if the ammonium is increased, we observe an initial phase where *P.putida* is growing and then the produced PHA is slightly lower (compare Fig. 3C and D).

Finally, the initial *P.putida* biomass does not directly correlate with the fitness, because it is inversely related with another parameter: NH_4_. Thus, when *P.putida* biomass is lower, sucrose is accumulated and is not completely transformed to PHA in 100 h (Fig. 3E). Then, NH_4_ should be increased because *P.putida* growth is required to get enough biomass to reach high PHA production (Fig. 3F).

### 3.3 Case study 2: Co-growth four auxotrophic *E.coli*

**Fig. 4.**
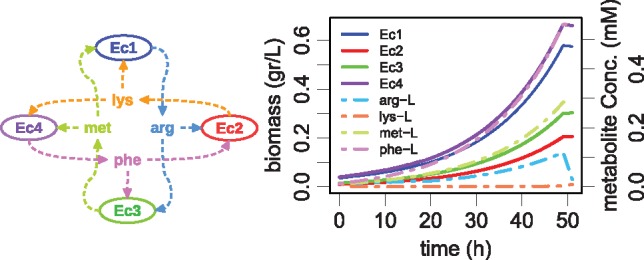
Description and FLYCOP solution for the co-growth four auxotrophic *E.coli* SteadyCom consortium. (**Left**) consortium description, adapted from (Chan *et al.*, 2017). **(Right)** dynamic growth curve showing cross-feeding amino acids and their accumulation evolution in the consortium designed with FLYCOP, with relative abundances: *Ec1 *=* *35%, *Ec2 *=* *10%, *Ec3 *=* *15%, *Ec4 *=* *40% and amino acid secretion rates (in terms of percentage of GR, with average 0.610 gDWh^−1^): arg = 1.5, lys = 2, met = 1.6, phe = 1

We selected this demonstration case to compare FLYCOP with the most recent consortia optimization method, SteadyCom (Chan *et al.*, 2017). The consortium to optimize is described in Figure 4-left: four *E.coli’*s with amino acids cross-feeding relations. Each strain uptakes from the medium the pair of amino acids that is unable to synthesize by itself, and secretes other ones. This domain illustrates a FLYCOP application of more than two strains consortium composition, optimizing parallel growth (i.e. stability) and configuring two types of parameters: strains ratio and cross-feeding rates.

We designed the single strains from the *i*AF1260 *E.coli* model (Feist *et al.*, 2007) according to the description and data provided in Table S2 from (Chan *et al.*, 2017) design, where the reactions and input amino acid uptakes are defined. Then, FLYCOP was applied resulting in the configuration whose dynamic simulation is illustrated in Figure 4-right. The FLYCOP effective GR is lower than that the one SteadyCom reached; whilst it proves FLYCOP is able to design the same type of consortium that SteadyCom and contributing with additional knowledge about how the microbial consortium dynamically works. This case study demonstrates FLYCOP can be applied to design microbial consortia requiring stability through similar GR in all strains, objective for which SteadyCom is limited.

FLYCOP reaches a community GR lower than SteadyCom (0.610 < 0.735 gDWh^−1^). Because of the extra carbon source consumed by synthesizing and secreting the amino acids involved in cross-feeding, a lower community GR than the wild type GR (0.737), as FLYCOP gets, makes physiological sense. In fact, we tested growing the four *E.coli* strains together in COMETS in a consortium with a manual configuration defined by the SteadyCom given strain ratios and adjusting amino acid secretion rates to reach the SteadyCom GR of 0.735 in each independent strain. Thus, it resulted in a co-living GR lower than the GR reported by SteadyCom, meaning their solution is out of FLYCOP solution space. This evidences SteadyCom does not take metabolite accumulation in the medium into account, assuming a direct transfer of amino acids from one strain to another. Thus, FLYCOP is based on other assumptions that allow the inclusion of more physiological knowledge about medium composition and dynamic change of metabolites, such as it is shown in the dynamic growth representation (Figure 4-right). Besides, FLYCOP optimized amino acid secretion rates (as proportion of GR): arg = 1.5; lys = 2; met = 1.5; phe = 1. SteadyCom did not make available their amino acids secretion rates to be compared.

There are alternative configurations that reach the same average GR values in the consortium, pointing out the robustness of that solution. All of them have common characteristics. First, in most of the alternative consortia configurations, arginine is the most limiting amino acid, explaining why relative abundance of its producer (*Ec1*) is high. However, in the best configuration, the most limiting amino acid is lysine (Fig. 4), even when the lysine secreting strain (*Ec2*) reaches its maximum secretion rate value. On the contrary, phenylalanine tends towards accumulation in all configurations. Thus, the predicted amino acids accumulation explains, at great extent, the lower community GR estimated by FLYCOP when compared with SteadyCom. Second, relative abundances follow the pattern: *Ec1+Ec4*: 75%/*Ec2+Ec3*: 25% (or 65/35%) versus SteadyCom: 50/50%. In FLYCOP solutions, the relative abundances ratios in decreasing order tend towards: *Ec1*, *Ec4*, *Ec2*, *Ec3*, with *Ec2* and *Ec3 *<* *25% each one and <50% together. The same relations *Ec1-Ec4* and *Ec2-Ec3* described in SteadyCom are evident with FLYCOP: one of each pair presents higher abundance, and that pair abundance is preserved in different good configurations, although the internal distribution of the relative abundances could change. Third, regarding the fitness correlation, a high fitness is correlated mainly with a high *Ec1* relative abundance (0.49), inversely with the *Ec3* relative abundance (−0.34) and after with lysine secretion (0.24).

According to FLYCOP results and data analysis of the multiple configurations, high values of arginine and lysine are required. We could explain this conclusion thanks to the dynamic simulation FLYCOP provides us showing how the metabolite concentrations change over time, which SteadyCom does not allow. Thus, community growth curves show that although those amino acids are finally accumulated, at the beginning they are very limiting and scarce nutrients (almost parallel lines to the x-axis), and therefore a lower secretion rate would not allow to start the community growth. Data analysis of explored configurations leads to conclusions about the range of values associated with good consortium configurations; for example, more than 50% of the evaluated consortia do not grow, being characterized by a low arginine and/or lysine secretion rate (<1).

Although the FLYCOP average GR is lower than the SteadyCom one, thanks to the use of a hybrid approach to model strains at a descriptive level by FLYCOP we can observe how dynamically the growth and the metabolite concentrations change in the medium, analyzing how they evolve and influence the global behavior of the community. Apart from strain ratios, the same as SteadyCom, FLYCOP also predicts the amino acid secretion rates.

### 3.4 Case study 3: Describing microbial community evolution

The third case study shows a different perspective of microbial communities, where FLYCOP also contributes, apart from design and optimization: to increase knowledge and understanding about microbial evolution; in other words, to find the biological objective of a microbial community that evolves over time. How? Through the selection of the objective function with the FLYCOP predictions most similar to *in vivo* experiments, among a set of different functions, each one providing a putative physiological explanation of the experiment.

To illustrate this new FLYCOP approach, we took the Long Term Experimental Evolution (LTEE) initialized in February 1988 by Richard Lenski (Lenski *et al.*, 1991). It consists on growing *E.coli* in serial cultures in flasks for a long time and check what happens. Cultures have been growing and passed everyday to fresh medium, and samples have been frozen periodically. After around thirty years, the Lenski’s group has cultured more than 60 000 generations, providing many samples, data and events to analyze bacterial evolution (Good *et al.*, 2017; Lenski, 2017).

Among the different events, we focused on a stable polymorphism appeared in population Ara-2 (one of the not growing on arabinose cultures, from the twelve ones in LTEE) (Rozen and Lenski, 2000). Over time, different mutations have emerged and accumulated if beneficial, appearing and disappearing several subpopulations; although only the Ara-2 has resulted in a stable polymorphism of two ecotypes, L and S. Those ones appeared around generation 6500, with morphological differences, with large (L) and small (S) colonies, respectively. Further knowledge about those two subpopulations have been known along diverse *in vivo* and *in silico* studies (Großkopf *et al.*, 2016; Lenski, 2017; Le Gac *et al.*, 2012; Plucain *et al.*, 2014). The S ecotype is able to grow more efficiently with a secondary product secreted during growth on glucose, which is acetate according to (Großkopf *et al.*, 2016), therefore we have decided to call glucose specialist and acetate specialist, respectively to L and S strains. L grows faster on glucose, and both ones faster than their ancestor.

Previous systems were also applied to LTEE domain: Inverse FBA (Zhao *et al.*, 2016) and evoFBA (Großkopf *et al.*, 2016). Inverse FBA characterized the objective functions compatible with measured fluxes, concluding that there are infinite ones could result in the observed fluxes, except to only maximizing GR, what is agree with FLYCOP results. evoFBA also studied LTEE with metabolic models. However, a quantitative comparison with FLYCOP is unfeasible, because none of both ones applied a community modeling approach, just a single species one, without considering the exchange of metabolites as FLYCOP does. A further textual comparison is available in Supplementary Material.

To represent this stable polymorphism in LTEE with FLYCOP, we took the *i*JO1366 *E.coli* model (Orth *et al.*, 2014), and we defined a wild type (WT) conditioned to a limiting carbon source (glucose), as in the LTEE experiment. We adjusted the WT model to physiological values of acetate secretion rate (3.7 mmol/gDWh^−1^) in glucose limitation conditions (10 mmol/gDWh^−1^ glucose uptake rate), according to (Steinsiek and Bettenbrock, 2012), where the uptake rates are available with a similar growth rate (0.8) to that one reported in LTEE experiments. FLYCOP began the search of the final polymorphism with a population of two equal WT strains at the same concentration (0.01 g/L of each strain). Then, FLYCOP checked the behavior of microbial communities composed by two *E.coli* strains with different uptakes and secretion rates of the three metabolites implicated in the heterogeneity (glucose, acetate and oxygen). In those conditions, FLYCOP searched the optimized community configuration given a specific biological goal. Due to that goal is unknown in this LTEE evolutionary process, we defined 5 different options, to solve in 5 different FLYCOP runs: three individual metrics to maximize (growth rate or biomass or yield) and two combined depending on time (to maximize biomass or yield in the minimum time). This case study combines next options of Table 1 FLYCOP applications: 2 homogeneous strains category, with different optimization goals including combinations of simple categories, configuring the parameters about co-metabolism: the carbon sources (glucose and acetate) and the oxygen uptakes of each strain.

**Fig. 5.**
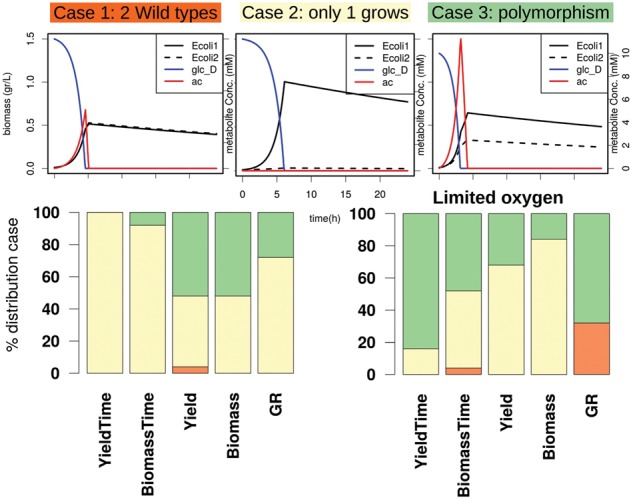
FLYCOP best consortium configurations profiles in LTEE with different fitness functions. Top: three representatives of the most common growth curves categories along different consortium configurations evaluated by FLYCOP. Bottom: cases frequency of 5% best configurations per fitness function (column names) which maximized a biological measure and sometimes also minimizes time

Figure 5 top row panel shows the most common growth curve profiles of consortia configurations evaluated with FLYCOP: 1) no differentiation with both strains the same as the wild type, 2) no differentiation where only one strain grows (with/out secreting acetate), and 3) polymorphism with two co-living strains growing first in glucose and after in previously secreted acetate. Each FLYCOP growth curve represents the final state after evolution.

As opposite to the previous case studies, FLYCOP found assorted best configurations. It means there are solutions with different profiles among those highest evaluated. This fact could represent that there are alternative consortia configurations leading to polymorphism, and one of them was successful in Ara-2 population; while other ones could have occurred, although not being persistent along the evolution (Barrick *et al.*, 2009). Nevertheless, to determine which fitness function better explains the Lenski’s experiment, we check in which one the highest frequency of polymorphism cases (green in Fig. 5) appears among the best configurations. Thus, maximizing yield or biomass are the biological goals FLYCOP suggests with more than 50% versus very low frequencies in other fitness functions (see Fig. 5, bottom row, left). Moreover, FLYCOP predicts that under environmental stress, such as limiting oxygen uptake (to 7 mmol/gDWh^−1^), the polymorphism frequency increases with different biological goals although being clearly dominant when yield is maximized and time minimized (Fig. 5, bottom row, right).

Therefore, the difficulty to find a robust polymorphism configuration points out this event of heterogeneous co-living is rare among the multiple appearances and deaths of slightly different strains along LTEE, what it is agree with experimental reports (Barrick *et al.*, 2009). In conclusion, despite it is a rare event, FLYCOP allow us to study multiple conditions and to select the most favorable one where polymorphism could emerge.

## 4 Discussion and conclusions

FLYCOP is a suitable tool for the *in silico* design of microbial consortia, allowing the simulation and detailed evaluation of different scenarios before facing up *in vivo* experiments with the definitive selected (optimal) configuration.

A limitation in current tools for designing microbial consortia with the hybrid modeling approach, is that they simulate one by one particular given scenario, not optimizing or selecting the best configuration, but only showing single snapshots, just at a descriptive level (such as COMETS). FLYCOP solves this limitation combining the hybrid approach with a further optimization phase.

FLYCOP is configurable to different and multiple applications of microbial consortia design. The application to the synthetic *S.elongatus-P.putida* consortium shows many of the FLYCOP capabilities to design and optimize consortia and how to use it given a new one. The co-growth four *E.coli* case of study compares FLYCOP with one of the most updated available methods to optimize consortia, i.e. SteadyCom, highlighting the flexibility of FLYCOP to optimize the same goal of stability (apart from others), and its additional capacity of dynamic flux control of the metabolites in the medium and the growth depending on it. Finally, the *in vivo* LTEE case of study evidences how FLYCOP could also be applied to study evolution in heterogeneous populations, suggesting the maximization of yield in minimum time as the most promising biological goal optimized when polymorphism emerges.

FLYCOP contributes in many aspects to *in silico* design and optimization of microbial communities. One of the most important contributions is the FLYCOP flexibility, because it is a common framework being applicable to very diverse microbial scenarios with distinct goals, computationally checking multiple community configurations. Moreover, different microbial consortium goals are easily changeable and their optimization results are comparable, in contrast to other current technologies limited to just a predefined goal such as stability. Varying the optimization goal is an important advantage, given that the growth rate could not be the best physiological option (Kreft *et al.*, 2017; Schuster *et al.*, 2008).

In addition, FLYCOP contributes with a decrease in experimental tests, guiding towards the most promising consortium configurations, saving up both resources and time. FLYCOP permits the integration of regulatory events in the community model, e.g. by switching reactions on and off or by tuning the bounds of fluxes in different single models, evaluating their effects in a dynamic way. FLYCOP helps to understand how microbial communities work at systems level. For example, illustrating what and when metabolites are accumulated or exhausted in the culture medium, and supporting with keys about how and why it happens, depending on the consortium evolution goal. In terms of descriptive method for metabolic modeling of microbial consortia, FLYCOP entails the hybrid or dynamic approach versus the multi-compartment or lumped network approaches selected by other frameworks that optimize microbial consortia based on metabolic models. This hybrid approach allows FLYCOP to describe temporal changes of metabolites concentration, and take dynamics into account, guiding the search of optimized consortium configurations using knowledge from simultaneous growth curves. FLYCOP facilitates an additional analysis of the assorted evaluated consortia and the combinations of configuration parameter values, with statistics and machine learning techniques, resulting in new knowledge retrievement and reasons explaining why the optimal configuration was selected versus others ones.

FLYCOP presents some limitations. First, the more parameters to be configured, the more time is required. Second, the descriptive hybrid approach (in what FLYCOP is based) is that allowing to represent more complex physiological properties of microbial communities. However, it also entails a drawback in terms of the number of strains in the consortium, being more limited than other engineered consortium modeling methods based on a different descriptive modeling such as multi-compartment one. Despite the limitation to low species richness, most of engineered synthetic consortia, it means the main FLYCOP application area, are composed of few strains.

Similarly to LTEE case study, a further FLYCOP application would be to predict the behavior of other long-term consortium with distinct strains, or even ‘domesticating’ evolution by modifying the final state after evolution by perturbing the environment under a nutrient limiting stress. Other interesting further work would be to apply FLYCOP to microbiome optimization, in a microbial community with available metabolic models, similarly to how SteadyCom does, taking the advantages of FLYCOP to induce changes in the medium and checking how it influences over community dynamics.

The importance of moving from the single strain engineering to the community engineering is remarkable given that opposite conclusions could come out modeling isolated strains versus modeling a microbial community with the modeled individual strains. For example, a particular strain could be the best one cleaning a metabolite pollutant at single strain modeling, while it could not be true when it would live in a community, because its new tasks for producing some cross-feeding products to stabilize the consortium could require more attention and energy than cleaning additional metabolites.

In conclusion, our metabolic-based computational framework to design, analyze and optimize consortia, FLYCOP, has a hopeful future contributing to the emerging field of engineering microbial communities; because those consortia are appearing as promising biotechnological tools to face up multiple current and coming applications and challenges in health and industry, even in new environments [such as living architectures (Armstrong *et al.*, 2017)] where microorganisms were not though as playing a fundamental role.

## Funding

This work was supported from the European Union's Horizon 2020 Research and Innovation Programme under Grant Agreement no 686585, and the Spanish Ministry of Economy and Competitivity through the RobDcode grant (BIO2014-59528-JIN).

*Conflict of Interest*: none declared.

## Supplementary Material

Supplementary Data
