# Supplementary material for: FLYCOP: metabolic modeling-based analysis and engineering microbial communities
Source: Bioinformatics. 2018 Sep 8;34(17):i954–63. doi: 10.1093/bioinformatics/bty561 (PMC6129290; doi:10.1093/bioinformatics/bty561)
Supplement: Supplementary Data [file bty561_supplementary_material.pdf]

Supplementary material:

FLYCOP: metabolic modelling-based analysis and engineering microbial communities

Beatriz García-Jiménez[1], José Luis García[2,3] and Juan Nogales[1,*]

[1]Systems Biology Department, Centro Nacional de Biotecnología (CNB-CSIC), 28049 Madrid, Spain, [2]Microbial and Plant Biotechnology Department, Centro de Investigaciones Biológicas (CIB-CSIC), 28040 Madrid, Spain and [3]Applied System Biology and Synthetic Biology Department, Institute for Integrative Systems Biology (I2Sysbio-CSIC-UV), 46980 Paterna, Spain

Contact: jnogales@cnb.csic.es

Materials and Methods

Brief description combined technologies

COBRA (Constraints Based Reconstruction and Analysis) (Schellenberger *et al.*, 2011; Ebrahim *et al.*, 2013) analyzes single-strain metabolic models. COBRA focuses on the application of the constraints imposed by the genome (internal boundaries) and environment (systems boundaries) in a given organism. It describes the set of feasible phenotypic states, in term of metabolic fluxes, under a given condition (Nogales, 2017). The constraint-based model consists of M metabolites and R reactions in an M x R matrix containing all the stoichiometric coefficients (**S**). Flux balance analysis (FBA) was used for the analysis, including growth and flux predictions (Orth *et al.*, 2010) FBA is based on solving a linear optimization problem by maximizing or minimizing a given objective function **Z** subject to a set of constraints. The constraints $\mathbf{S}\cdot\mathbf{v} = \mathbf{0}$ correspond to a situation of steady-state mass conservation where the change in concentration of the metabolites as a function of time is zero. The vector **v** represents the individual flux values for each reaction. These fluxes are further constrained by defining lower and upper limits for flux values. Gurobi 6 solver were used for solving the linear programing problems in the current described FLYCOP applications.

COMETS (Computation Of Microbial Ecosystems in Time and Space) (Harcombe *et al.*, 2014) combines different metabolic models to design and simulate interactions in microbial consortia in a dynamic context (time and/or space). COMETS is a hybrid model (Perez-Garcia *et al.*, 2016) that simulates dynamic of metabolite and biomass of multiple strains in the same physical space. The temporal dynamic of this hybrid model is simulated with the updates of the shared media metabolite concentrations and the update of biomasses, according to the metabolic activity of the organisms in the community. COMETS allows to update the metabolite concentrations with different algorithms. The default approach is to update each metabolite multiplying the uptake by the biomass of each strain secreting or consuming that metabolite. The biomass of each strain within the consortium is predicted following the same algorithm in COBRA, i.e. Flux Balance Analysis (FBA), although with some differences. For instance, it runs along several consecutive time slots in a similar way to a dynamic FBA, but it uses a dynamic computation of the bounds of the exchange reactions taking into account the updated concentration of the corresponding metabolite in the media in

each time slot, which could be lower than the original bound in the model. Many other parameters could be configured, following the available documentation in the COMETS website (http://www.bu.edu/segrelab/comets).

SMAC (Sequential Model-based Algorithm Configuration) (Hutter et al., 2011) allows to automate a model parameterization with an iterated local search, guided by ad-hoc fitness. Stochastic local search is one of the most efficient and widely used methods for solving combinatorial problems, which are characterized with an exponential or higher (such as NP) complexity, where approximate solutions are often useful and easier to compute. It allows exhaustive exploration of big state spaces to be avoided, finding good solutions more efficiently than systematic search; although these kinds of algorithms could be incomplete, this means they do not guarantee to find a solution even if one exists. There are different stochastic local search methods (simulated annealing, tabu search, etc.), where the best are the hybrid ones, i.e. combinations of several simple search strategies (such as iterated local search or evolutionary algorithms).

At the beginning, SMAC establishes an initial (default) configuration as a solution. The best solution found is preserved until a better one is reached (i.e. lower fitness, since SMAC minimizes the fitness value) or the algorithm ends; when the pre-defined number of iterations or timeout is reached. In each iteration, random modification of some of the parameter values are applied; first, with small modifications, searching in the neighborhood of the new solution. Where a better configuration is not achieved after several iterations, the algorithm then applies large changes in the values, trying to go out from a local minimum, and exploring a distant area in the solution space. Specific details about condition-specific SMAC setups can be found in the proper Results sections. The problems solved with FLYCOP described in this manuscript applied SMAC v2.10.

Detailed comparison consortia optimization methods

FLYCOP vs optimizing community parameters (OptCom (Zomorrodi and Maranas, 2012) and d-OptCom (Zomorrodi *et al.*,2014)). OptCom allows modeling or evaluation of consortia, not suggesting automatic configuration as FLYCOP does. OptCom requires several executions with the initial compound concentration in each time slot, versus FLYCOP capturing temporal changes. This dynamic limitation was solved with its improved version framework d-OptCom. (d)OptCom optimizes in two levels, the inner level (single strains) with the parameter optimized in the outer level (community). However, FLYCOP optimizes consortia configuration at different levels at the same time, both at single strain level (i.e. uptakes of carbon sources or cross-feeding metabolites) and community level (i.e. initial ratio biomasses and after n hours, total biomass at different time points, metabolite concentration in the media by time slot). Besides, FLYCOP offers flexibility in the parameters to configure (integer, real or even nominal ones). d-OptCom also uses dFBA to optimize fluxes. Regarding simplicity, FLYCOP does not configure any MILP problem with new reactions for new interactions among strains, nor to known the type of interactions, as (d)OptCom requires. Our approach only needs the individual models and the media composition, and the required interactions will emerge by themselves based on metabolism codified in models. The complementary 'Descriptive d-OptCom' is equivalent to include parameters about the media and initial biomasses in FLYCOP. Regarding experimental kinetics data requirement, one d-OptCom input is the uptake kinetics parameters independent per metabolite (or they assume

direct transfer of metabolite between input and output through fluxes), while in FLYCOP it is optional, according to COMETS configuration (which allow define the same kinetics parameters for all metabolites). Thus, FLYCOP could work with less experimental data that d-OptCom, that requires uptake kinetics parameters independent per metabolite. Besides, we could represent the problem in order to find some optimal uptakes in FLYCOP. Finally, d-OptCom is defined as a 'comprehensive computational framework', not providing any software that allows reproducibility or new applications as FLYCOP does.

FLYCOP vs optimizing stability (SteadyCom (Chan *et al.*, 2017)). The main difference with FLYCOP is related to flexibility in objective and use-cases, focusing SteadyCom on consortia where the GR must be the same in all members in the community, to ensure co-existence. FLYCOP could optimize consortia with that objective of parallel GR (through defining a fitness function to minimize the difference in GR in exponential phase) among other multiple ones. SteadyCom joins all reactions in a common model, being more similar to lumped network than multi-compartment modeling approach. Apart from FBA, SteadyCom is compatible with FVA. Both ones provide with a similar contribution in the cross-cutting task of predicting composition on microbial consortia. Other similarity is the ability to predict changes in strain relative abundances in response to external perturbations (such as changes in media metabolites) although through distinct approximations: SteadyCom by adding constraints on the model and FLYCOP by including static or dynamic modifications in the media in COMETS configuration file, along the time (or in some specific time point) or running a new optimization with a new media composition or media update. FLYCOP intrinsically explores multiple combinations of model parameters, making them available for further analysis; while SteadyCom requires an external procedure when they want to analysis random consortium configurations.

FLYCOP vs optimizing pathway distribution (MultiPlus (Julien-Laferrière et al.,2016)). In contrast to the previous approaches, MultiPlus does not optimize a set of parameters of the metabolic models or community properties as FLYCOP and the remaining ones do, but optimize the fragmentation of a pathway and distribution of the reactions among the species within the community, even selecting the subset of strains. This means it is constrained to be applied in only one use-case (distributing pathway), only one optimization goal (minimizing new reactions and minimizing exchanged metabolites), not flexible in both as FLYCOP. Pathway distribution approaches are usually stoichiometric-independent, simplifying GEM to a representation in an hyper-graph, with just reactions and metabolite relations. It means a drawback versus other approaches because it results in unchecked consortium configurations which could not growth together when it is constrained to stoichiometry, requiring post-processing steps to compute yield (proposing to use a posteriori FBA) and to get stability of the consortium or even including not defined cross-feeding relations; whilst FLYCOP reports multiple values for measuring the performance of the community (GR, increase in biomass, yield, etc.), without requiring additional post-processing, and additionally providing the optimized GEMs as an output, allowing their run together in a microbial community descriptive microbial community system such as COMETS, showing common grow and production of metabolites, and checking if some cross metabolite is danger to the other strains. It is similar to FLYCOP in terms of searching a solution among different configurations. The same as MultiPlus, FLYCOP also could optimize the production of endogenous metabolite, not only exogenous. Regarding to optimizing distribution of reactions, maybe it could be better to apply some of the existing regardless-stoichiometric graph-based methods (Eng and Borenstein, 2016; Julien-Laferrière et al.,2016) to reduce the

huge number of possibilities, and between the best outputs, to apply FLYCOP taking the stoichiometry and the consortium dynamic (updates of biomass and metabolite concentration) into account to finally define the optimized distribution of tasks among the strains in the consortium.

LTEE case study: comparing FLYCOP with inverseFBA and evoFBA

Inverse FBA (Zhao et al., 2016) also was applied to this LTEE domain to determine the objective function. Their most relevant conclusion for FLYCOP is that there are infinite objective functions could give the observed fluxes as one of the optimal solutions of an FBA, and only maximizing growth rate is not included in those solutions. Many solutions tend towards strains with higher respiration flux (~= glucose specialist) and other solutions with lower respiration (~= acetate specialist). They did not infer a separate set of fluxes of both strains in the Ara-2 population, but only one set, assuming it is a monoclonal population, ignoring the stable polymorphism. In fact, the fluxes for Ara-2 are more similar to glucose specialist (with slight higher growth than ancestor) than acetate specialist.

evoFBA (Großkopf et al., 2016) is an alternative work about LTEE, randomly generating different niches of specialized species along the time, assuming different phenotypes in the same strain, the same as we consider. evoFBA runs the evolution and finds a solution consortia at the end, resulting from intermediate mutations along the evolution. Whereas FLYCOP defines different consortia configuration a priori (it means to apply some mutations apriori) and checks whether its viability and stability along time, finding the best consortium configuration for a predefined goal. evoFBA uses a reduced *E.coli* metabolic model with 95 reactions, while we use the whole model with thousands of reactions, although the uptake mutations is only in one or a few, we do not remove the remaining model reactions because all are involved in the *E.coli* metabolism. evoFBA only runs isolated strain models, not simulating co-culture of the acetate-glucose specialists as FLYCOP does.

Both methods predict the cross-feeding among two *E.coli* specialists as a stable end-point after evolution. Both systems have two optimization levels: a) FBA and b) evolutionary time-scale (in FLYCOP with SMAC). At the evolutionary one, evoFBA constraints the overall input uptakes (carbon and oxygen) per organism, to simulate in a simplified way the trade off observed in in-vivo studies; however, FLYCOP allows flexible “mutations” in the model depending on the parameters you want to determine, given an optimization goal. evoFBA predicts a completely loss of taking acetate in L strains, although invivo data it is really a down-regulation, as FLYCOP allows. evoFBA could determine when the mutant strain appears, while FLYCOP pre-defines it exists, without knowing when it will appear during the evolution. FLYCOP conditions/mutations are only defined at the beginning of the consortia growing point, without changes in uptakes during time, as evoFBA allows. Their mutations are in each time steps along the evolution, not just as the beginning like we do.

A further results comparison is unfeasible, because evoFBA does not simulate co-culture of the acetate-specialist with glucose-specialist, but they only can run dFBA for isolated strains/models.

References

Chan, S. H. J., Simons, M. N., & Maranas, C. D. (2017). SteadyCom: Predicting microbial abundances while ensuring community stability. PLoS Computational Biology, 13(5), e1005539. https://doi.org/10.1371/journal.pcbi.1005539

Ebrahim, A., Lerman, J. A., Palsson, B. O., & Hyduke, D. R. (2013). COBRApy: COnstraints-Based Reconstruction and Analysis for Python. BMC Systems Biology, 7(1), 74. https://doi.org/10.1186/1752-0509-7-74

Großkopf, T., Consuegra, J., Gaffé, J., Willison, J. C., Lenski, R. E., Soyer, O. S., & Schneider, D. (2016). Metabolic modelling in a dynamic evolutionary framework predicts adaptive diversification of bacteria in a long-term evolution experiment. BMC Evolutionary Biology, 16(1), 163. https://doi.org/10.1186/s12862-016-0733-x

Harcombe, W. R., Riehl, W. J., Dukovski, I., Granger, B. R., Betts, A., Lang, A. H., … Segrè, D. (2014). Metabolic Resource Allocation in Individual Microbes Determines Ecosystem Interactions and Spatial Dynamics. Cell Reports, 7(4), 1104–1115. https://doi.org/10.1016/j.celrep.2014.03.070

Hutter, F., Hoos, H. H., & Leyton-Brown, K. (2011). Sequential Model-Based Optimization for General Algorithm Configuration (extended version). Retrieved from https://www.cs.ubc.ca/~hutter/papers/10-TR-SMAC.pdf

Julien-Laferrière, A., Bulteau, L., Parrot, D., Marchetti-Spaccamela, A., Stougie, L., Vinga, S., … Sagot, M.-F. (2016). A Combinatorial Algorithm for Microbial Consortia Synthetic Design. Scientific Reports, 6(1), 29182. https://doi.org/10.1038/srep29182

Nogales, J. (2017). A Practical Protocol for Genome-Scale Metabolic Reconstructions. In Hydrocarbon and Lipid Microbiology Protocols : Genetic, Genomic and System Analyses of Pure Cultures (pp. 197–221). https://doi.org/10.1007/8623_2014_12

Orth, J. D., Thiele, I., & Palsson, B. Ø. (2010). What is flux balance analysis? Nature Biotechnology, 28(3), 245–248. https://doi.org/10.1038/nbt.1614

Schellenberger, J., Que, R., Fleming, R. M. T., Thiele, I., Orth, J. D., Feist, A. M., … Palsson, B. Ø. (2011). Quantitative prediction of cellular metabolism with constraint-based models: the COBRA Toolbox v2.0. Nature Protocols, 6(9), 1290–1307. https://doi.org/10.1038/nprot.2011.308

Zhao, Q., Stettner, A. I., Reznik, E., Paschalidis, I. C., & Segrè, D. (2016). Mapping the landscape of metabolic goals of a cell. Genome Biology, 17(1), 109. https://doi.org/10.1186/s13059-016-0968-2

Zomorrodi, A. R., & Maranas, C. D. (2012). OptCom: A Multi-Level Optimization Framework for the Metabolic Modeling and Analysis of Microbial Communities. PLoS Computational Biology, 8(2), e1002363. https://doi.org/10.1371/journal.pcbi.1002363

Zomorrodi, A. R., Islam, M. M., & Maranas, C. D. (2014). d-OptCom: Dynamic Multi-level and Multi-objective Metabolic Modeling of Microbial Communities. ACS Synthetic Biology, 3(4), 247–257. https://doi.org/10.1021/sb4001307
